# Effect of Oxygen Sputter Pressure on the Structural, Morphological and Optical Properties of ZnO Thin Films for Gas Sensing Application

**DOI:** 10.1007/s11220-017-0184-5

**Published:** 2017-12-29

**Authors:** S. Fairose, Suhashini Ernest, Samson Daniel

**Affiliations:** 1 PG & Research Department of Physics, Urumu Dhanalakshmi College, Trichy, Tamil Nadu 620 019 India; 2 PG & Research Department of Physics, Bharathidasan University, Trichy, Tamil Nadu 620 024 India

**Keywords:** Ammonia, Chemiresistor, Sputtering, Sensitivity

## Abstract

ZnO thin films were prepared on glass substrates at low (5 × 10^−4^ mbar) and high (3 × 10^−3^ mbar) sputter pressure using dc reactive magnetron sputtering. The structural, morphological, compositional and optical properties of the thin films were investigated. XRD patterns of both films confirmed the polycrystalline nature of the films with hexagonal Wurtzite structure. SEM study indicates that the surface of the film formed at high sputter pressure was more uniform, compact and porous in nature. From the EDAX analysis, no other characteristic peaks of other impurities were observed and the formation of single phase of ZnO was confirmed. From the study of photoluminescence, three peaks were observed, one strong near band-edge emission at 390 nm followed by weak and broad visible emissions around 420–480 nm. Room temperature ammonia sensing characteristics of ZnO nanothin films formed at higher sputter pressure were studied for different ammonia vapour concentration levels. The response of the Ammonia sensor at room temperature (30 °C) operation was observed to be of high sensitivity with quick response and recovery times.

## Introduction

Semiconducting gas sensors play vital role in detecting, monitoring and controlling the presence of ammonia vapour in the atmosphere at very low concentrations, which is hazardous to humans [1]. It is used in various fields such as industrial, environmental, automotive and in domestic application for pollution control and safety purposes [2]. The current occupational safety and health administration’s (OSHA) permissible exposure limit (PEL) for ammonia is 35 ppm at a 15-min short term exposure limit (STEL). The limits are based on the risk of eye irritation and respiratory effects associated with exposure to ammonia. Ammonia vapour is severely irritating to the eyes and to the moist skin and mucous membranes of human [3, 4]. When liquid anhydrous ammonia comes in contact with the eyes, it may cause severe damage such as blindness [5]. Hence, there is a need for ammonia detection for diagnosis, water and waste water analysis.

Various techniques are available to detect ammonia such as catalytic ammonia sensors [6, 7], optical gas sensors [8] and spectrometric ammonia detection [9], which are expensive and need experienced operators. But chemiresistive sensors are simple, portable, stable, reliable and highly selective while exhibiting high accuracy with faster response and recovery times [10].

Numerous efforts have been made with doped/mixed ZnO nanostructures to detect the NH_3_ concentration in air atmosphere at room temperature [11–13]. But from the literature, it is seldom to find undoped ZnO thin films as NH_3_ sensors at room temperature with better response and selectivity [14–16]. Since ethanol, toluene, methanol, acetone, benzyl alcohol and acetic acid are the most common interfering gases in indoor air atmosphere [17, 18], the ability to fabricate thin films insensitive to other gases in the presence of NH_3_ is important.

Attempts have been made to detect ammonia by using ZnO hierarchical structures such as, ZnO nanowire, nanorods, nanofibers, nanoflakes, nanosheets, nanoplates and nanothin films [53–59]. Among these morphologies, the present work (nanoparticles) exhibit better sensitivity with quick response and recovery time.

Stability of the ammonia sensor plays an important role in developing gas sensors for different application [19]. Mani et al. reported about the long term stability of the sensing characteristics of ZnO films were tested over a period of 12 days towards 25 ppm of ammonia vapour at dry air atmosphere and 52% relative humidity. The same trend was confirmed quite a lot of times and hence the reproducibility factor was also proved.

Cui et al. [20] reported about fabrication of resistance based Ag nanocrystal-functionalized multiwalled carbon nanotubes (Ag NC-MWCNTs) sensor and discussed NH_3_ sensing properties namely, sensitivity, superfast response and recovery, and good stability at room temperature [20]. Singh et al. [21] discussed about the reversible conductivity change in the opposite manner on exposure to ppm levels of H_2_S and NH_3_ gases of the sensor photopolymerisation of pyrrole using AgNO_3_ as a photo-initiator. Joshi et al. [22] investigated the NH_3_ sensing properties of Polypyrrole thin films gas sensors at room temperature fabricating by chemical polymerization method and also reported the selectivity, sensitivity with linear response in range of 4–80 ppm.

Joshi et al. [23] analysed the hierarchical NiCo_2_O_4_ structure based gas sensor for the detection of Ozone and measured the sensitivity of the sensor at 200 °C temperatures with fast response (32 s) and recovery (60 s) time with suitable concentration range (from 28 to 165 ppb). Navale et al. reported the importance of NO_2_ vapour sensing property of ZnO nanorods (NR’s) and bunch of nanowires (BNW’s) fabricated by thermal evaporation method. And also discussed about sensing response of 622 and 101%, along with rapid response and recovery times, to toxic NO_2_ gas for ZnO NR’s and BNW’s respectively [24].

Several techniques were used to get a quality film such as sol gel method [25], SILAR [26], spray pyrolysis [27], CBD [28] and thermal evaporation [29]. Among these methods, sputtering is one of the important techniques that has several advantages such as (1) low substrate temperature (down to room temperature); (2) good adhesion of films on substrates; (3) very good thickness uniformity and high density of the films and (4) directive deposition from elemental (metallic) targets by reactive sputtering in rare/reactive gas mixtures [30]: the stoichiometry of the films can be controlled easily and its high deposition rates onto a large area offers good control over the composition of the film [31].

Zinc Oxide (ZnO) is one of the wide band gap semiconductor materials [32] with large excitonic binding energy (60 meV) [33]. It has large surface area for gas adsorption/desorption compared with other materials. It is a non toxic material [34] having high transmittance at visible range [35]. A room temperature ammonia sensor based on high sputter pressure ZnO nanothin films can adsorbed and desorbed the NH_3_ molecules with enormously which leads to reduce the operating temperature, increase in the sensitivity, quick sensing response and recovery times. In the present study, ZnO films are prepared by using dc magnetron reactive sputtering and their structural, optical and morphological properties for vapour sensor application are investigated.

## Experimental Details

### Synthesis of ZnO Nanostructures

The Zinc Oxide films were deposited onto cleaned glass substrates (glass slides of 75 mm × 25 mm × 1 mm) by dc reactive magnetron sputtering method. A stainless steel vacuum chamber, 300 mm in diameter and 370 mm height was pumped using diffusion pump–rotary pump combination with a liquid nitrogen trap, which could give an ultimate vacuum of the order of 10^−6^ mbar. The pressure was measured using a Pirani–Penning gauge combination. A circular planar magnetron with 70 mm diameter erosion zone was used as a cathode. A continuously variable dc power supply of 750 V and 3 A was used as power source. The sputtering target was 99.99% pure metallic zinc (obtained from Nuclear Fuel Complex, India) of 100 mm diameter and 3 mm thick. High purity (IOLAR-1 grade) argon and oxygen were used as the sputtering and reactive gases respectively. The flow rates of both the argon and oxygen gases were controlled individually by using Tylan Mass Flow Controller Model FC-260. The oxygen pressure (pO_2_) was maintained at 5 × 10^−4^ mbar (low pressure) and 3 × 10^−3^ mbar (high pressure) and the sputtering pressure was maintained at 6 × 10^−2^ mbar by controlling the flow of argon. The target to substrate distance was maintained as 65 mm. Before deposition of an oxide film, the Zinc target was resputtered in an argon atmosphere for about 15 min in order to remove the surface oxide layer of the target.

### Characterization

X-ray powder diffraction (XRD) is recorded on Holland Philips Xpert diffractometer (Cu Kα = 1.5418 Å). Scanning electron microscopy (SEM) and energy dispersive spectroscopy (EDX) of synthesized ZnO samples is recorded by a Holland Philips XL30 microscope instrument to investigate the morphology as well as the elemental composition of the sample. The surface structure of the films was examined by an ex situ atomic force microscopy (AFM). The AFM images were acquired in the contact mode and in the repulsive force regime. The optical characteristics of the thin films were observed using photoluminescence (PL) measurements are taken at room temperature using He–Cd laser line of 275 nm as the excitation source.

### Sensor Fabrication and Testing Method

**Fig. 1:**
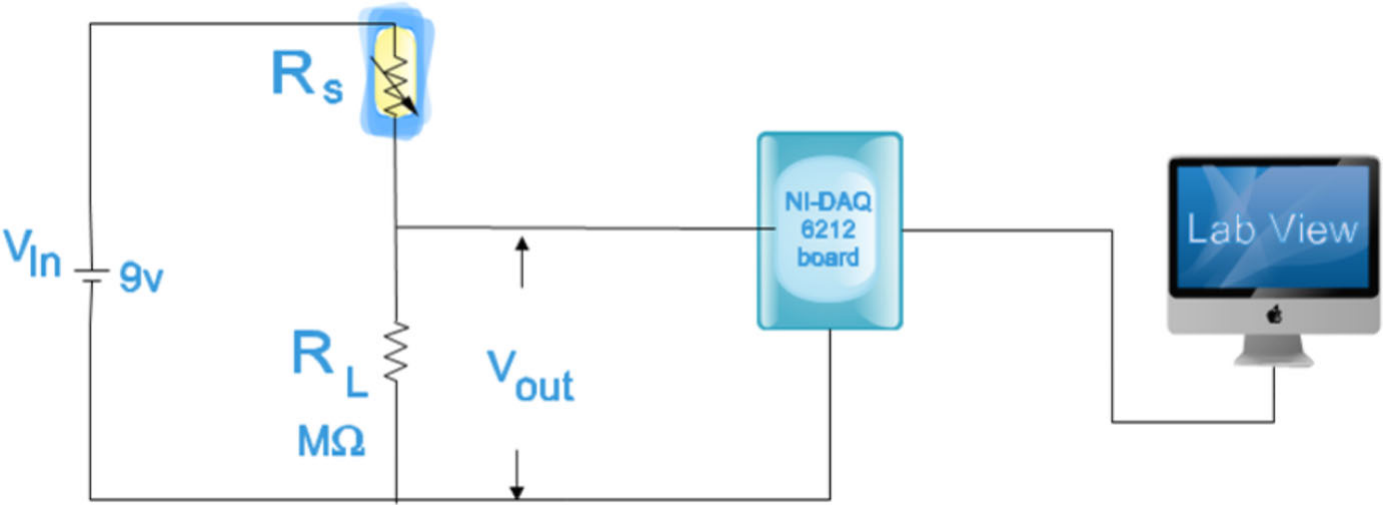
The schematic circuit diagram of the gas sensitivity measuring unit

The vapour sensing properties of the film have been studied using home-build test chamber of 1.5 L capacity [36]. Before sensing studies, the film was conditioned at 300 °C for 24 h to remove undesirable pre-adsorbed organic and water molecules. Ohmic electrical contacts were made on the film (12 mm × 10 mm) using thin copper wire and silver paste. Fabricated sensor was placed in the test chamber filled with room air having relative humidity of 20% and a known quantity of dry ammonia. All the measurements were recorded at room temperature (i.e., 30 °C). Variation of real time voltage signal across the resistance connected in series with the sensor was recorded with an experimental setup consisting of NI-DAQ Data Acquisition Module 6212 with Lab-VIEW software and is converted into electrical resistance shown in Fig. 1. The sensor response magnitude was determined as R_g_/R_a_ ratio, where R_g_ and R_a_ were the resistances of sensor in air–gas mixture and air ambience.

## Results and Discussion

### Structural Properties

**Fig. 2:**
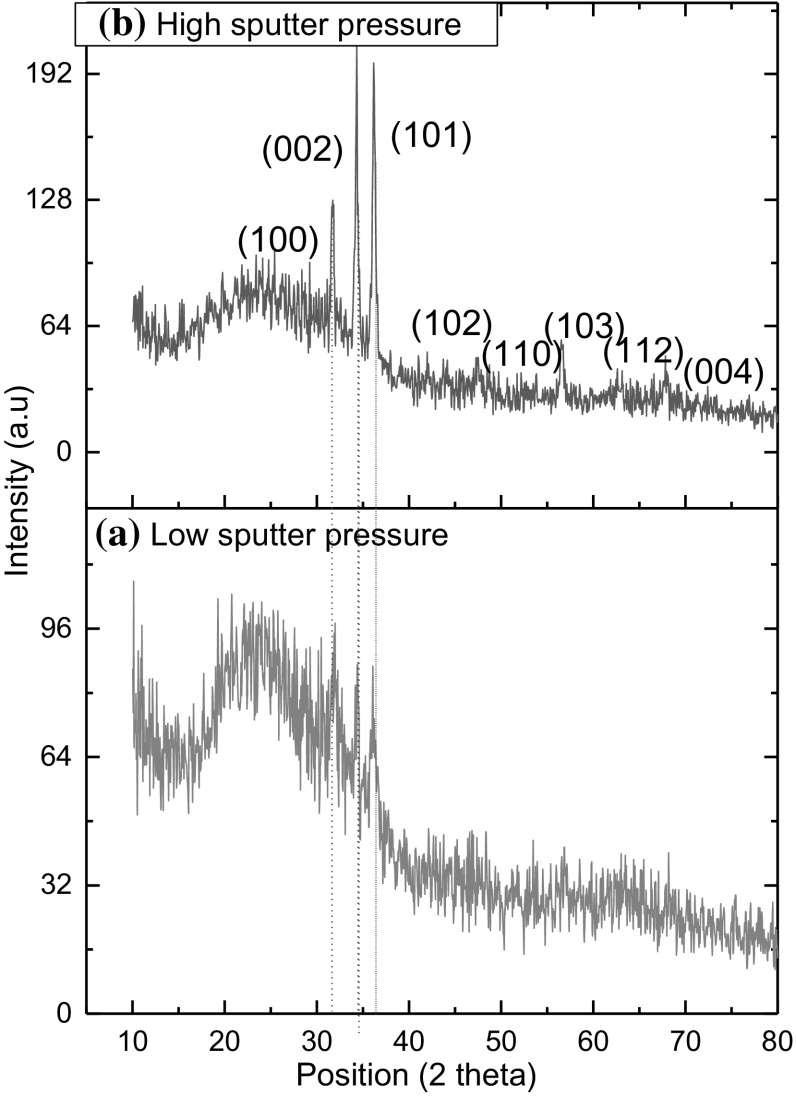
XRD patterns of ZnO films grown at room temperature at (**a**) low and (**b**) high oxygen sputter pressures

Figure 2a, b show typical X-ray diffraction (XRD) pattern of the ZnO nanothin films at low (5 × 10^−4^ mbar) and high (3 × 10^−3^ mbar) sputter pressures. XRD indicates that the oxygen sputter pressure affects the deposition of ZnO films. Figure 2b has diffraction peaks at 31.68°, 34.32°, 36.09°, 47.7°, 56.5°, 62.5°, 67.8°, and 72.79° corresponding to lattice planes of (100), (002), (101), (102), (110), (103), (112) and (004) respectively. The diffraction peaks can be indexed to ZnO (JCPDS no. 36-1451), consistent with the data reported in the literature [37]. XRD spectra were recorded in the 2ϴ range 10°–100°. However, the diffraction peaks were observed between 10° and 80°. The presence of diffraction peaks indicates that the films are polycrystalline in nature. From the absence of impurity peaks in XRD results, the phase purity of the ZnO is inferred. The XRD pattern of thin film formed at low sputter pressure shown in Fig. 2a is observed to have few peaks at 31.68°, 34.32° and 36.09° corresponding to lattice planes of (100), (002) and (101) respectively. Hence, the film formed at high sputter pressure is preferred for investigating the morphological, compositional and sensing properties.

The distinct crystalline peak seen in XRD for the film formed with high sputter pressure can be attributed to the hexagonal Wurtzite structure indexed to the (1 0 1), (0 0 2) and (1 0 0) planes with respect to the standard JCPDS 36-1451. Reduction in defect as well as improved crystallinity was obtained for ZnO film formed with high pressure. This aids the adsorption of ammonia vapour molecules on the surface of the film when used for sensing application. Hence, the film formed at high sputter pressure is preferred for investigating the morphological, compositional and sensing properties.

The lattice constants ‘a’ and ‘c’ calculated by using the following Eqs. (1) and (2) are given in Table 1 [38].

**Table 1:** Variation of lattice parameters, crystallite size and stress of ZnO films as a function of sputter pressure

ZnO thin film	a (Å)	c (Å)	Crystallite size D (nm)	Stress (GPa)
Low pressure	3.023	5.222	15.73	− 71.60
High pressure	2.997	5.212	53.29	− 26.85

1 $$ a = \sqrt {\frac{1}{3}} \frac{\lambda }{\sin \theta } $$

2 $$ c = \frac{\lambda }{\sin \theta } $$

In addition, the calculated lattice parameters are in close agreement with standard diffraction pattern value (36-1451).

The residual stress (σ) is calculated by the following formula, which is well adopted for a hexagonal lattice [39].

3 $$ \sigma = \frac{{2c^{2}_{13} - c_{33} (c_{11} + c_{12} )}}{{2c_{13} }}\frac{{c - c_{0} }}{{c_{0} }} $$

In which the elastic constants *c*_ij,_ values for ZnO thin film are used as given below *c*_11_  =  208.8; *c*_33_  =  213.8; *c*_12_  =  114.7 and *c*_13_  =  104.2 [39].

Sputtering pressure has strong influence on stress value of ZnO thin film [30]. At lower oxygen pressure, the ZnO film exhibits − 71.60 GPa, which decreases with increase in oxygen pressure, leading to a − 26.85 GPa for film formed at high pressure, indicating a reduction in defect.

The crystallite size is calculated using Debye–Scherrer formula [40].

4 $$ D = \frac{k\lambda }{\beta \cos \theta } $$

where D is the diameter of the crystallite, λ is the wave length of the CuKα line (λ = 1.5418 Å), β is the full width at half maximum in radians and ϴ is the Bragg angle.

Table 1 presents the grain size of the films which increases from 15.73 to 53.29 nm with the increase of oxygen pressure from 5 × 10^−4^ to 3 × 10^−3^ mbar. Thus a reduction in defect as well as improved crystallinity was obtained for ZnO film formed at high pressure. This aids the adsorption of ammonia vapour molecules on the surface of the film when used for sensing application.

### Surface Analysis

#### SEM and EDAX Analysis

**Fig. 3:**
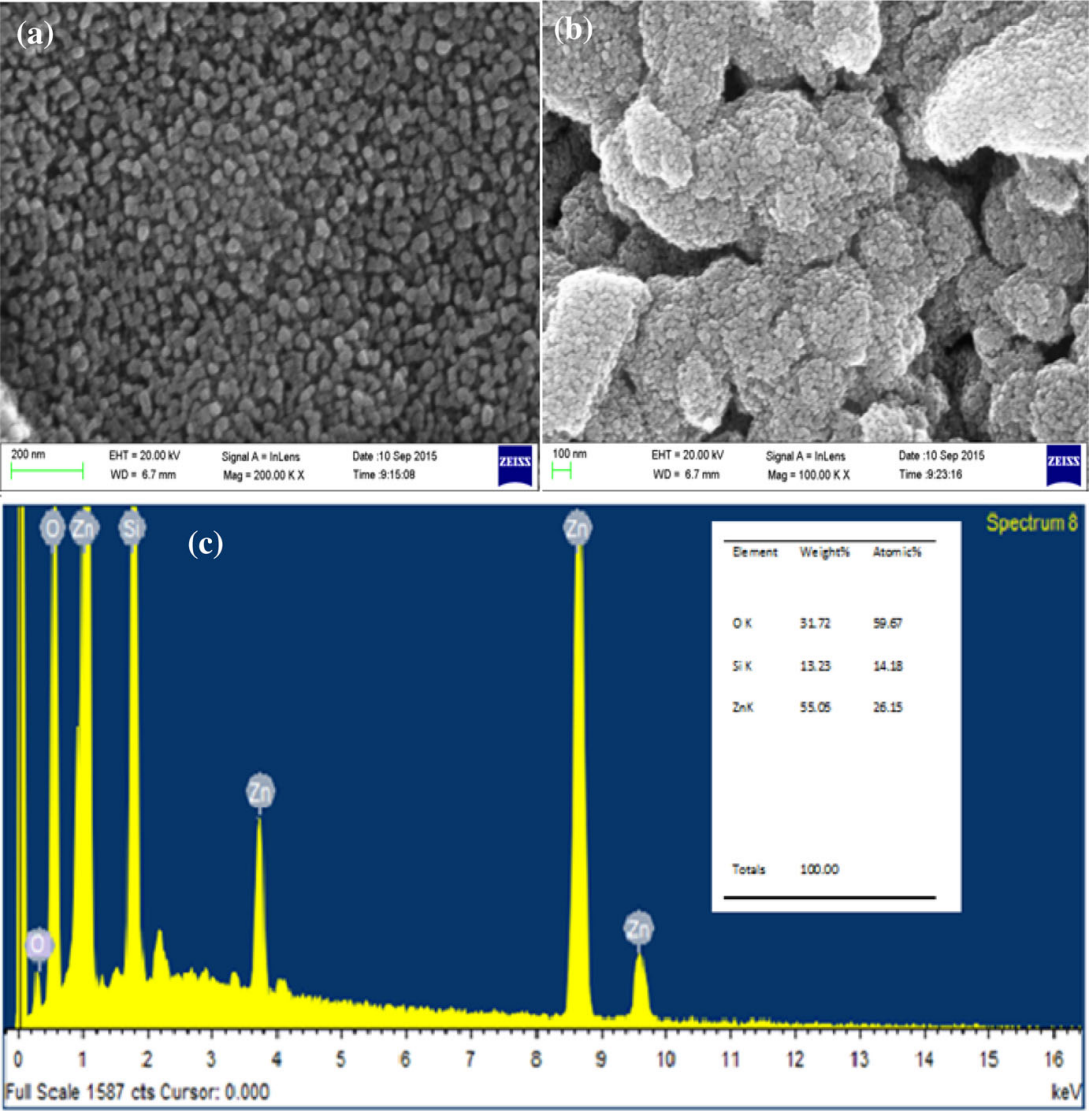
**a**, **b** Typical scanning electron microscopy (SEM) images of ZnO (at high pressure) nanothin films with different magnifications **c** energy dispersive X-ray spectroscopic spectrum of ZnO nanothin films formed at higher sputter pressure

Figure 3a, b depict the SEM images of ZnO films formed at high oxygen sputter pressure at different magnifications. The SEM images show the ZnO nanothin films and their uniform distribution over the substrate, the shape and size of these nanoparticles being confirmed by XRD results. An energy dispersive X-ray (EDAX) spectrum of the ZnO thin film nanoparticles is shown in Fig. 3c. It is clear that there are no impurities from other materials. The silicon signal appears from the substrate and the level of silicon contamination is indicated. The weight percent of Zn and O element obtained from EDAX is given in the inset figure.

#### AFM Analysis

**Fig. 4:**
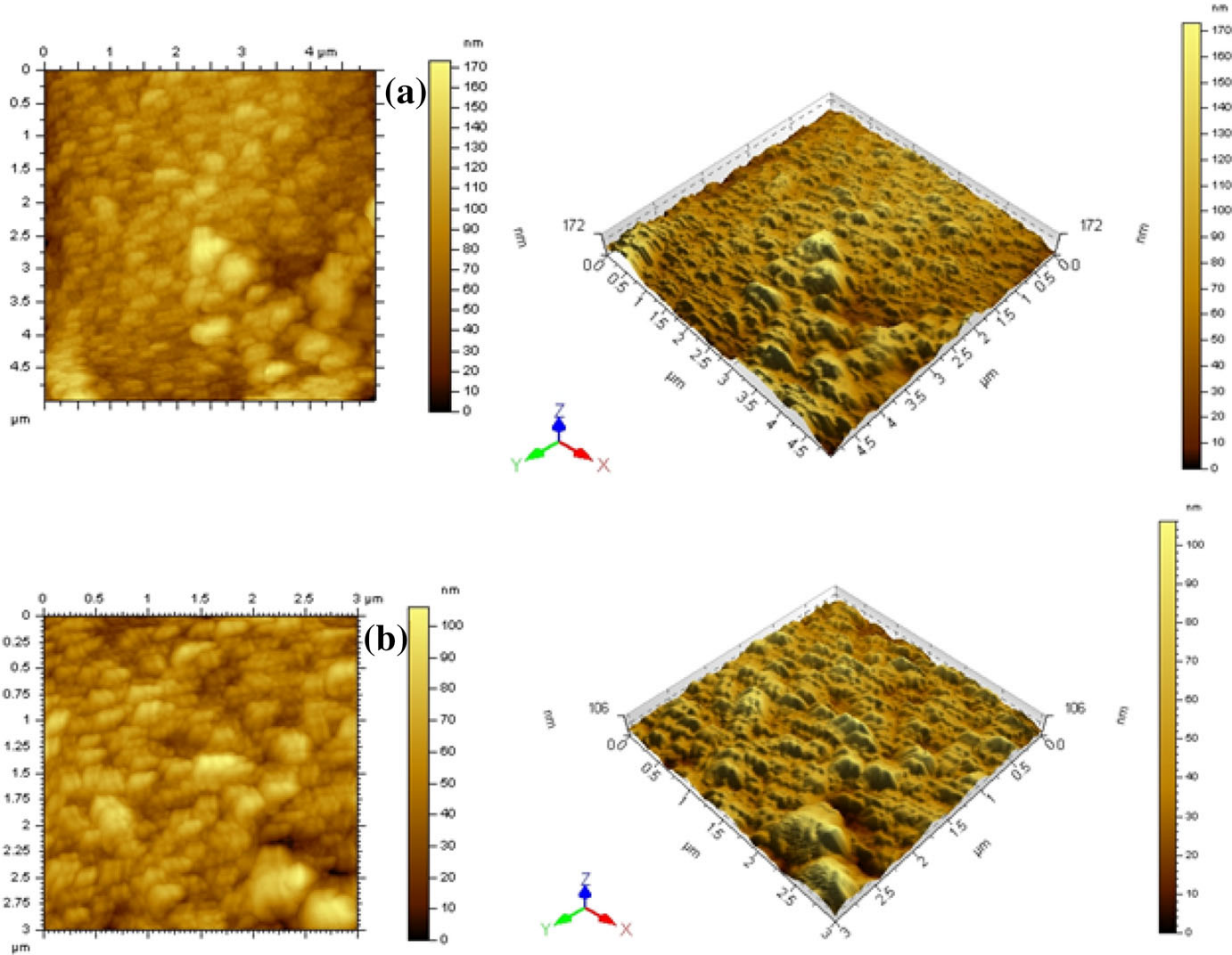
**a**, **b** Atomic force microscopy (AFM) images of ZnO thin films at low and high sputter pressure

Figure 4a, b indicates the AFM images of ZnO films formed at low and high oxygen sputter pressure. The comparative study of films formed at low and high sputter is made only in the topographical studies. Film formed with high sputter pressure showed uniform spherical morphology when compared with low sputter pressure. Similar behaviour is reported in other research paper [19].

### Optical Studies

#### Photoluminescence

**Fig. 5:**
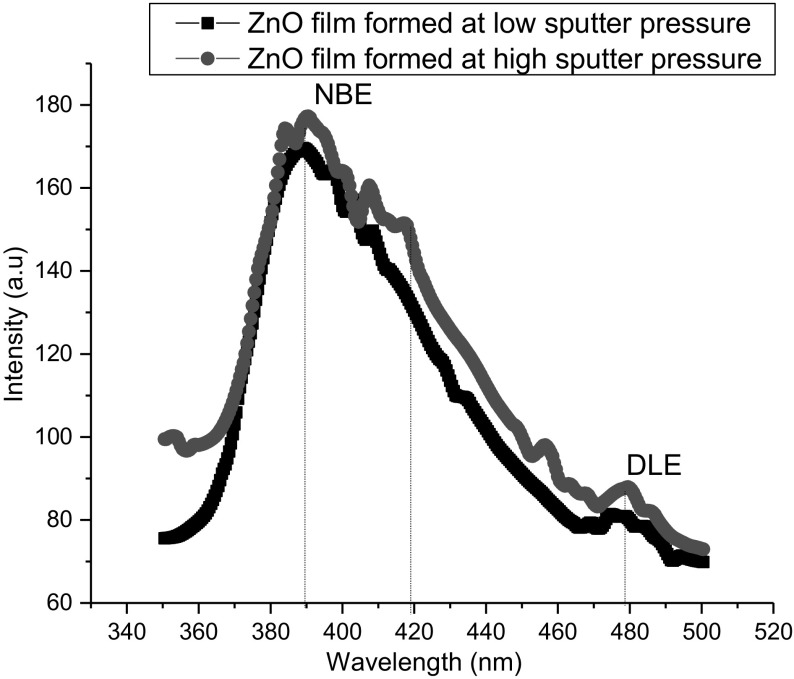
The photoluminescence spectra of ZnO films under different oxygen sputtering pressures

The room-temperature photoluminescence spectra of nanocrystalline ZnO films prepared under different sputtered pressures at room temperature are shown in Fig. 5. It can be seen that three emission bands were observed in both the samples such as (1) UV emission (< 400 nm) band, (2) violet emission (420 nm) band, (3) blue emission (480 nm). The samples exhibit strong near band-edge emission (NBE) at 390 nm followed by a weak and deep level emission around the visible region 420–480 nm. NBE originates from free-exactions recombination through an exciton–exciton collision process (so called P line) or electron–hole plasma (EHP) [41], whereas the visible emission attributed to the deep level emission (DLE), originates from the exciton recombination in the localized states. The shape of both spectra, being similar to those reported by Kandasamy and Lourdusamy [41], is featured by a strong emission near UV and a defect-related deep level emission in visible region. The UV emission originates from free excitonic emission [42]. Exciton recombination between the electrons localized at the zinc interstitials and holes in the valence band leads to this violet emission. In addition to violet, blue emission has been observed sharply around 480 nm in both samples. From the present study, it is clear that increase sputtered pressure increase of thin film size is favourable for the violet emission and it widens the UV emission range from 399 to 417 nm.

### Vapor Sensing Studies

For ZnO thin film a semiconductor material, the chemical reactions occurring on the surface lead to the absorption of oxygen from the atmosphere. This adsorbed oxygen gets electrons from the conduction band of ZnO and is converted into oxygen ions (O^−^ or O^2−^). This reaction is shown in the Eq. (5).

5 $$ {\text{O}}_{2} + {\text{e}}^{ - } \mathop \to \limits_{{\begin{array}{*{20}c} {surface} \\ {of} \\ {\text{ZnO}} \\ \end{array} }}  {\text{O}}_{2}^{ - } (ads) $$

**Fig. 6:**
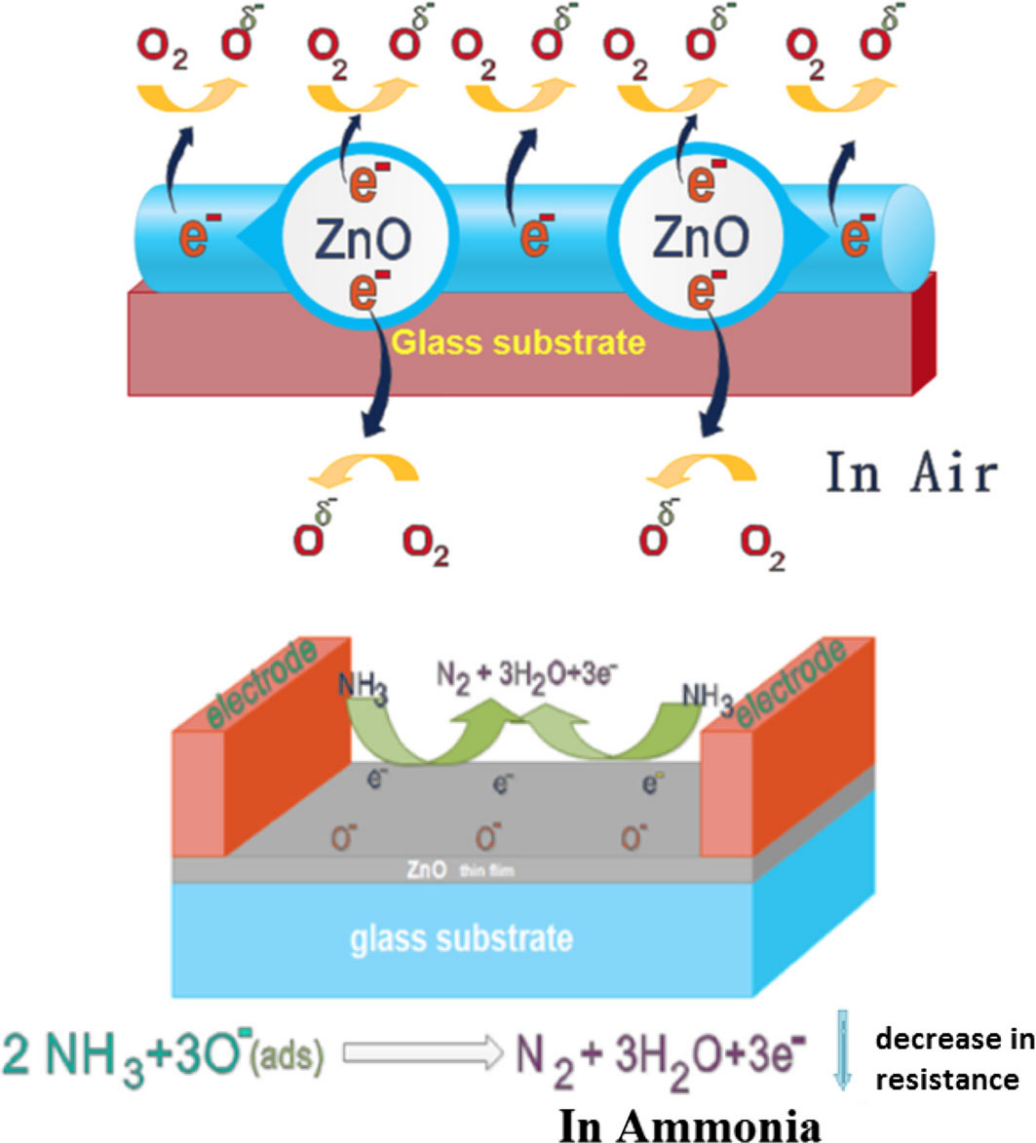
Schematic diagram of the sensing mechanism in the presence of air and ammonia

These results in the decrease of carrier concentration due to formation of depletion layer in the surface of ZnO thin film, which in turn explains the high resistance of the film observed in the ambient air condition. When target gas like ammonia is introduced on the film surface, the adsorbed oxygen ion reacts with NH_3_ vapour, resulting in the reduction of the sensor resistance, as the captured electrons are released to the conduction band of ZnO thin film. Therefore the conductivity of the ZnO sensors is increased [43]. Equation (6) gives the reaction on the introduction of ammonia vapour. And a possible schematic diagram of air and ammonia vapour detection mechanism is given in Fig. 6.

6 $$ 2{\text{NH}}_{3} + 3{\text{O}}^{ - } \left( {ads} \right) \to {\text{N}}_{2 } \uparrow + 3{\text{H}}_{2} {\text{O}} \uparrow + 3{\text{e}}^{ - } \downarrow in\;resistance $$

#### Resistance Response

**Fig. 7:**
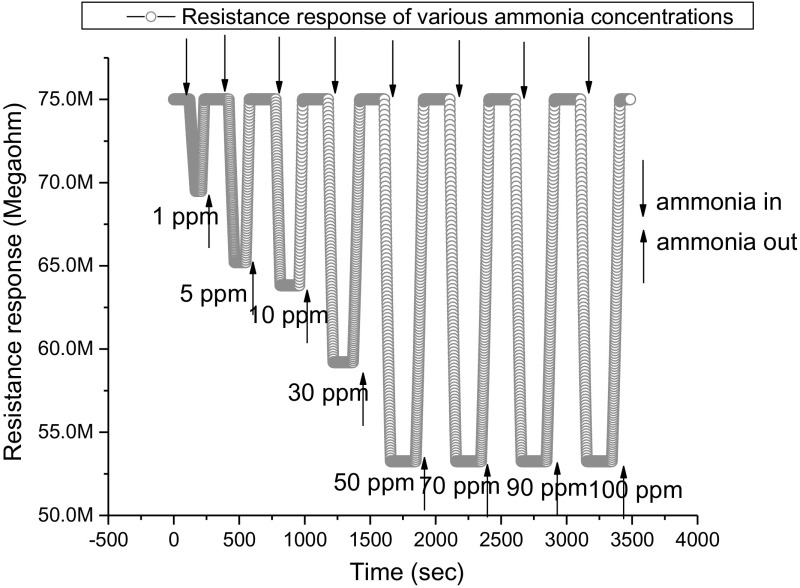
Variation in the resistance of the ZnO film (coated at high sputter pressure) for various concentrations of ammonia vapour

ZnO thin film resistance response is explained for film formed at high sputtering pressure. The sensor resistance decreased in the presence of NH_3_ vapour, due to the decrease of oxygen on the ZnO film surface. The sensing properties of ammonia vapour sensor were studied at room temperature. The resistance response of various concentrations of ammonia vapour at room temperature is shown in Fig. 7.

#### Sensing Response

**Fig. 8:**
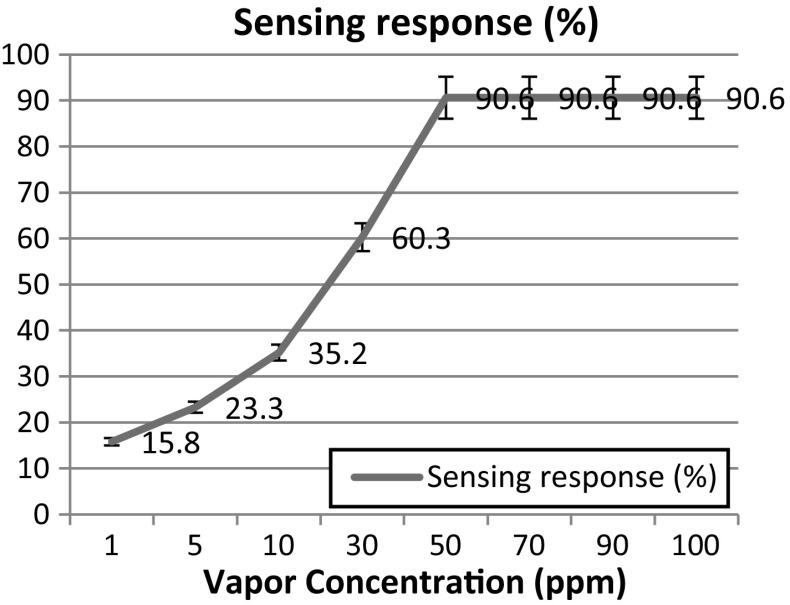
Sensing response of the ZnO thin film coated at high sputter pressure for different concentrations of ammonia vapour

The sensing response is expressed according by the following Eq. (7) [44].

7 $$ S\% = \frac{{R_{0} - R_{g} }}{{R_{g} }} \times 100\% $$

where R_0_ is the resistance of the sensor in the presence of air and R_g_ is the resistance of the sensor in the presence of ammonia. From the electrical resistance response curve of ZnO nano thin film to different concentrations of NH_3_ at room temperature given in Fig. 8, the sensing response is calculated to be 15.8, 23.3, 35.2, 60.3 and 90.6 for 1, 5, 10, 30 and 50 ppm of NH_3_ vapour respectively. An appreciable increase in response was observed as the ammonia concentration is increased from 1 to 50 ppm and this good response is attributed to the smaller grain size, improved crystallinity and the large surface area of nano structured film as discussed in the structural studies. When the concentration of ammonia vapour was increased beyond 50 ppm, no appreciable change in the resistance was observed.

#### Response and Recovery Time

**Fig. 9:**
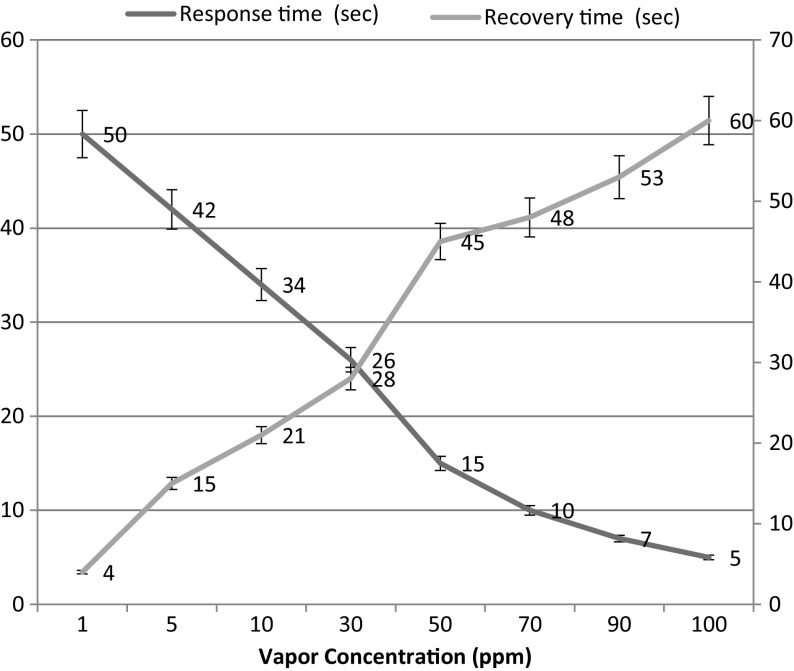
Variation in response and recovery times of ZnO thin film coated at high sputter pressure at different ammonia concentrations

**Fig. 10:**
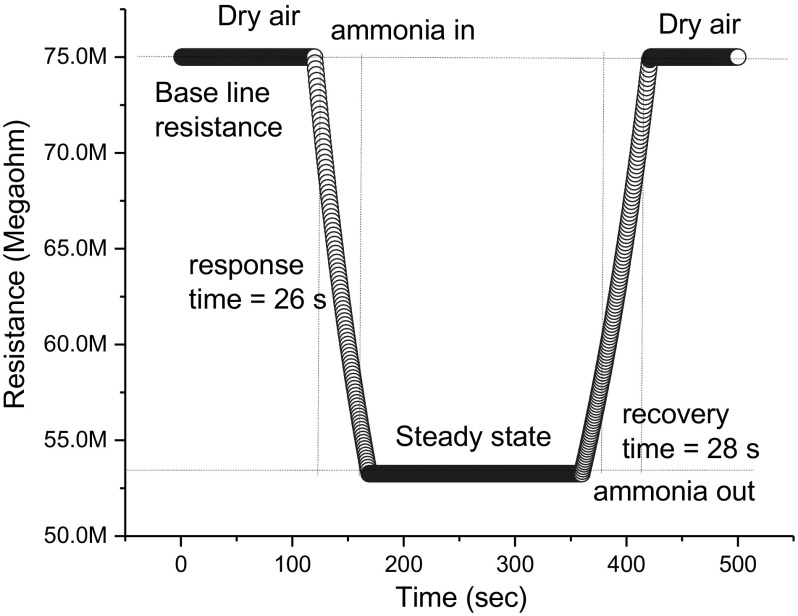
Transient resistance response of ZnO thin film towards 30 ppm of NH_3_

The resistance response of the ZnO thin film formed at high sputter pressure in dry air and in the presence of NH_3_ (at various concentrations) is measured. The response and recovery times for each concentration of NH_3_ vapour is tabulated in Table 2 and represented in Fig. 9. The response time is defined as the time required for the film to reach a 90% decrease from the baseline resistance when introducing ammonia and recovery time is defined as the required time needed for the sensor to attain base line resistance from the steady state resistance after removal of ammonia vapour [45]. When a high concentration of reducing gas like ammonia vapour is exposed on the ZnO surface, the large amount of NH_3_ molecules react with adsorbed oxygen O_2_^−,^ and hence a shorter response time is observed; the adsorbed molecules take a longer time to desorbs on the removal of NH_3_ vapour, which in turn increases the recovery time. In contrast, for lower concentrations of NH_3_, less ammonia molecules are adsorbed on the ZnO surface leading to a slow response, resulting in longer response time. And the lower vapour concentration was found to have faster recovery time since the interaction of chemisorbed ion with ZnO nanoparticles is weak, Fig. 10 depicts that the quick response and slow recovery time observed at 30 ppm ammonia vapour concentration.

**Table 2:** Sensing parameters with various ammonia vapour concentrations

Vapor concentration (ppm)	Response time (s)	Recovery time (s)	Sensing response (%)
1	50	4	15.8
5	42	15	23.3
10	34	21	35.2
30	26	28	60.3
50	15	45	90.6
70	10	48	90.6
90	7	53	90.6
100	5	60	90.6

**Table 3:** Comparison of ZnO thin film with various metal oxide heterostructures with respect to ammonia sensing properties

Other metal oxide heterostructure	Detection range (ppm)	Sensor response (%)	Response time (s)	Operating temperature (°C)	References
PANI/SnO_2_	100	29	33	Room temperature	[46]
ZnO–rGO	50	2.38	107	25	[47]
TeO_2_	500	58	181	170	[48]
Pd^2+^–ZnO	75	30	275	200	[49]
Pd/SnO2/rGO	100	20	420	25	[50]
PANI–TiO_2_	20	~12.5	62	25	[51]
PANI–SnO_2_	100	72	198	25	[52]
WO_3_–ZnO	300	2500	60	250	[53]
ZnO	50	90.6	15	Room temperature	Present work

The room temperature response of ZnO nanothin films at high sputter pressure for 50 ppm of ammonia was found to be high (S = 90) with faster response and recovery times of 15 and 45 s respectively (Fig. 9). The observed result is compared with other available results of various Metal oxide heterostructure thin films [46–53] and is presented in Table 4 as well as compared with other available results of various nanostructure ZnO thin films [53–59] and is presented in Table 3.

**Table 4:** Comparison of ZnO thin film with various nano structures with respect to ammonia sensing properties

Structure	Detection range (ppm)	Sensor response (%)	Response time (s)	Recovery time (s)	Operating temperature (°C)	References
Nano flakes	250	182	1200	1200	175	[54]
Nano sheets	50	263	8	14	250	[55]
WO_3_–ZnO nanoplates	300	2400	60	50	250	[53]
Nano rod	10	–	7	9	Room temperature	[56]
Nano flower	50	45.7	20	60	100	[57]
Nano fibres	100	2.5	–	–	Room temperature	[58]
PPy/ZnO nanosheets	5	155	256	370	Room temperature	[59]
WO_3_–ZnO nanorod	300	2500	60	50	250	[53]
Nano thin film	50	90.6	15	45	Room temperature	Present work

**Fig. 11:**
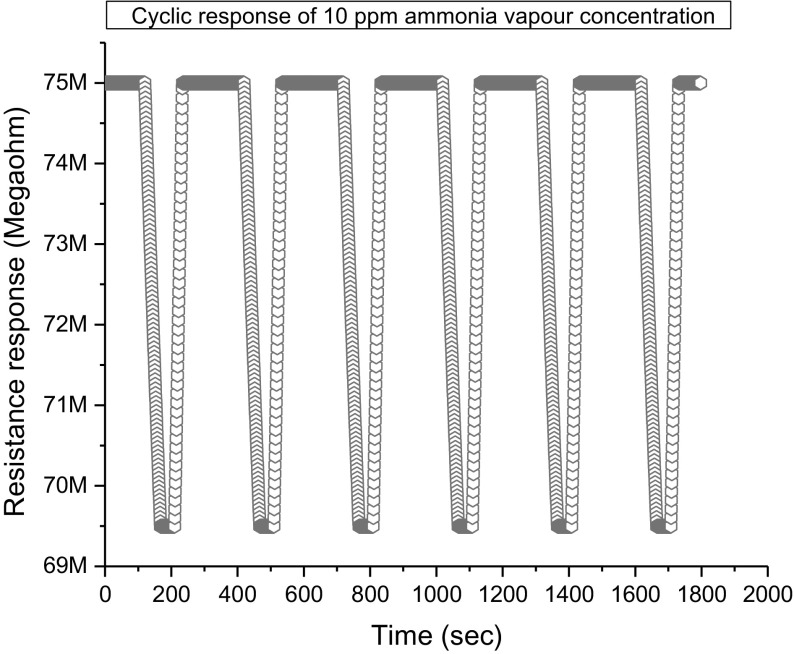
The cyclic resistance response towards 10 ppm of NH_3_

The cyclic response (repeatability) of the ZnO film for 50 ppm studied at constant intervals is shown in Fig. 11. Establishing the fact that the repeatable redox reactions are taking place on the surface of ZnO.

## Conclusion

ZnO nanothin films were grown on the glass substrate using the dc reactive magnetron sputtering method. The considerable change in the resistance observed at various ammonia concentrations ranging from 1 to 50 ppm indicates the better sensing behavior of the nanocrystallite of ZnO thin film. A highest response of 90.6 was achieved at 50 ppm of NH_3_ because of the increase in the interaction between the ZnO thin film and ammonia vapor, and quick response and recovery times of 26 and 28 s was obtained at 30 ppm. The lowest detection limit was found to be 1 ppm. The observed results suggest that ZnO film can be employed to detect ammonia vapor at room temperature for environmental, water and waste water analysis.
